# Medical and Physical Intelligence

**Published:** 1821-03

**Authors:** 


### Medical and Physical Intelligence.

---

TRANSACTIONS OF SCIENTIFIC SOCIETIES.

ROYAL SOCIETY *of London.—Jan.* 18. A PAPER by Dr. DAVY was read, giving an account of his inquiries relative to the urinary organs, and secretion of two species of common rana in Ceylon: from which it appears, first, that the bladder of the bull-frog and brown-toad (the two species in question) is a genuine receptacle of urine, which it receives from the cloaca, in which the ureters terminate; and secondly, that their urine is not at all analogous to that of other animals of the order amphibia, being very dilute, containing urea and certain salts, but no appreciable quantity of lithic acid. This peculiarity of urine, so well adapted to the size and structure of the bladder, is the more remarkable, as the favourite food of these animals is the same as that of small lizards, whose urine is of a butyraceous consistence, and nearly pure lithic acid. Hence, and from other facts mentioned by the author, he adduces the conclusion that the nature of urine, in every instance, depends much more on the peculiar action and structure of the secreting organs, than on peculiarities of diet or of the circulating fluids.

---

Dr. Fouquier, of Paris, to whom we owe the introduction of *nux vomica* into use, has lately been trying the efficacy of another powerful remedy—the *acetate of lead*, when given in larger doses than those in which it had hitherto been administered. "When a physician must renounce all hope of curing his patients," says Dr. Fouquier, "there often yet remains for him this consolation, that of alleviating their sufferings and prolonging their existence." The profuse sweats in phthisis contribute much to exhaust the patients of that disease, and hasten the fatal termination, especially when accompanied with colliquative diarrhœa. Dr. Fouquier thinks that he has found in the acetate of lead a mean of repressing this debilitating evacuation. He first employed that medicine in the solid form, in pills of a grain each; but he has since found it more convenient to administer it in solution, in a mucilaginous mixture. He relates the histories of twelve cases, arrived at the last stages, in which this medicine was used, in one to the extent of twelve grains as a dose, without finding after death, in the patients who died in the hospital, any affection of the intestines which might be attributed to the mineral. In one or two individuals only were some slight colic pains manifested; and these did not appear to be owing to the medicine, as they were accompanied with diarrhœa, and did not vary whether or not the use of it was continued. In a few cases there was constipation, for the removal of which glysters became necessary.

---

We give following abstracts of two cases, related with several others of more or less interest, by Dr. Venturi, first physician *della città di Sanseverino*, that seem to us to present some very interesting facts for the consideration of theoretical pathologists, as well as medical practitioners.

A girl, twenty years of age, was, in 1812, suddenly affected with very severe rheumatic pain in the upper and middle part of the back. A physician prescribed a spirituous embrocation. When this had been used two or three times, the pain disappeared from its first seat, and fixed just about the union of the last dorsal and first lumbar vertebræ. The use of the embrocation was continued; but the pain soon became almost insupportable, and seemed to be deeper seated. After a few days' very severe suffering, the patient began to experience a sense of formication in the lower limbs, that was soon followed by a sense of weight, and then, gradually, complete loss of sense and power of motion in them. Dr. Venturi saw her first after the lapse of four months from the occurrence of the paraplegia. The patient had then a *chlorotic* aspect, and had not menstruated for four months; the urine and feces passed unconsciously and involuntarily; and the lower limbs were flaccid, emaciated, insensible, and motionless. A dull and deep pain remained in the part of the spine above designated. The pulse became accelerated, and presented a vibrating character, towards the evening, when there was an increase of heat over the surface of the body generally, excepting in the paralytic limbs; the temperature of which was always below the natural standard. A gentle sweat came on towards morning, with which the fever disappeared. There was a periodical discharge of a large quantity of mucus from the uterus, accompanied with a sense of heat in the part, as if the uterus were affected with a sort of catarrh. Dr. Venturi commenced the cure by the abstraction of about ten ounces of blood from the back, in the region of the part affected, by means of leeches, and the administration of three grains of kermes mineral, in six doses, daily, which were accompanied by a draught of infusion of arnica-flowers. After three days, the state of the patient having suffered no change, the dose of the kermes mineral was doubled, and two scruples of mercurial ointment were rubbed along the spine. This friction was repeated nineteen times, in thirty-four days. The warm bath was also employed. After the fifth friction with the ointment, the patient became conscious of the passing of her urine and feces, and in two or three days more the evacuation of them was entirely under the dominion of the will. After the ninth friction, the patient felt pain about the great toe of the right foot, like that from a recent blister: this sort of pain then extended over the foot, and was soon followed by a return of sensibility to external impressions over the whole limb. Two days afterwards, the return of sensibility occurred, in a similar way, in the left extremity; and in a few days more, the power of motion in both of them. The mercurial inunction was now omitted. Some occasional painful spasmodic contractions of the affected limbs now occurred, which were the only inconveniences experienced by the patient. These soon subsided, under the use of the warm bath and the infusion of arnica. It was thought proper to treat the patient for another month with steel medicines and infusion of gentian; at the end of which time not the least vestige of the disease remained.

The other case above alluded to, was one of *amaurosis*.

A youth, thirteen years of age, of a slender habit of body, had been subject, from time to time, from his infancy, to ophthalmia. In the year 1813, he had a new attack, but slight to external appearance, and confined to the right eye, but accompanied with deep and very acute pain, which ceased during the whole of the night, but returned towards morning, and continued without intermission till the evening. During the paroxysms of pain, the affected eye appeared unusually injected with blood, the tears flowed copiously; the temporal artery on that side beat with violence, and the corresponding venous ramifications were tumid. At first there was intolerance of light, but afterwards the eye became more and more insensible in this respect; and on the lapse of about two months the sight of the affected eye was entirely lost, the pain having ceased, and no other morbid appearance of the eye being manifest than a little inordinate dilatation of the pupil. At this time the same inflammatory symptoms occurred in the other eye, and it was feared that they would be followed by similar results, and, consequently, that total blindness would ensue; the stimulus of light producing at length but a feeble impression on the eye secondarily affected. Such was the state of the patient when he was first seen by Dr. Venturi, which was five months from the commencement of the disease. Many and opposite kinds of medical measures had been employed; as purgatives, sarsaparilla, china-root, valerian, ether, musk, camphor, opium, various collyria, vesicatories to the arms, and a seton in the neck.

Dr. Venturi remarked a great disposition to morbid excitement about the head: a slight degree of bodily exercise was sufficient to produce great heat in it, with copious sweating. He determined to treat the case by sedatives. Ten leeches were applied about the temple and external angle of the left eye. During the paroxysm of pain, a pledget wetted with iced water, frequently renewed, was kept on the eye. A spare diet was ordered; and the internal administration of oxide of zinc and extract of cicuta, in quantities of four grains of the former and eight of the latter daily. The quantities were gradually increased to fifteen grains of the zinc and twenty of the cicuta: but then, after a little time, vomiting being excited, they were reduced to the original extent. The paroxysms of pain soon ceased, and left the eye last affected in the natural state. At this time a favourable change took place in the right eye; the sense of sight began to reappear, and it was after two months perfectly re-established. The patient presented for some time the curious phenomenon of a transudation of blood in the spot occupied by the cicatrix of the seton, whenever the excitement in the head, above mentioned, took place.

---

We have received the following account of two interesting operations from Mr. Richard Walker, of Oxford.

The modern operation for the cure of aneurism by the application of a single ligature, has been very adroitly performed here lately in two instances: one, in which the external iliac artery was tied for an aneurism of the femoral artery, toward the upper part of the thigh; and another, in which the femoral artery was tied for the popliteal aneurism.

The first subject, being an old man of sixty, in a very weak emaciated state, promised but small hopes of success: his life, however, I may venture to say, has been preserved by the operation, the aneurism being in a very advanced state; but the circulation was too languid to preserve the leg, which was amputated below the knee; a direct line of separation between the living and dead parts having there taken place.

The latter instance, being in a healthy young man, succeeded completely, without the occurrence of the least untoward circumstance. The ligature became perfectly detached and was taken away on the twentieth day. The aneurismal tumor is much diminished, and the patient enjoys the perfect use of his limb, which is free from sensations of numbness or coldness.

I mention these cases, occurring in a provincial situation, principally to inculcate additional confidence, if needful, in such operations, being well assured, from my knowledge of operations in general, that, in instances of the former kind, provided the subject be a tolerably fair one, the operation being judiciously performed, with due attention afterwards, there can be little doubt of complete success; neither mortification nor hemorrhage from ulceration being to be apprehended. In cases of the latter kind, under the before-mentioned favourable circumstances, scarcely any doubt of success.

It unfortunately happens that some persons are particularly predisposed naturally to aneurism: such is the case in the latter instance; there being, at the time of the operation, an aneurism, in a recent state, in the other ham: but this was justly considered no sufficient objection to the operation; which will probably be required, and some time hence may be performed, for this affection in the other ham.

N.B.—It is well known that, in the old way of operating for the popliteal aneurism, mortification and death were so commonly the consequence, that, latterly, amputation in the first instance was deemed the better alternative.

I have ventured thus promptly, as a professional spectator, to transmit the above account of these operations: it is not improbable that a more circumstantial account of the case in which the iliac artery was tied may, some time hence, appear from the operator himself.

---

A Journal has just been established at Paris, by Dr. Magendie, "*for Experimental Physiology.*" With such a title, however, the Number that has been published contains articles of a strictly anatomical nature, and some histories of cases of diseases, as well as papers relative to vegetable chemistry and pharmacy. There existed already an excellent Journal for Physiology, (the *Deutsch Archiv fur die Physiologie* of Professor Meckel,) which comprises papers collected from the literature of Europe in general, as well as original articles by the conductors; but that of Dr. Magendie is intended, it appears, to contain only original papers; and, as he has taken up the cultivation of this part of medical science with so much zeal, he may continue, as he has done in the Number that has been published, to supply his Journal to a great extent by the results of his own researches. There are several very good articles in the present Number; and, as it may be interesting to our readers to know with what auspices this Journal has made its first appearance, we shall give an abstract of the whole of its contents; but we have vacant space left in the present Number only for one of the shortest articles, which is that relating a novel experiment respecting *rabies* in the dog. After an account of the notions that have been entertained of the nature of this disease, Dr. Magendie expresses some doubts of the success of any attempts to cure it by ordinary means, for the following reasons. "The most active substances, the most powerful narcotics, have no action on either man or animals affected with rabies." "I do not speak merely of substances introduced into the stomach, the action of which might be prevented or attenuated by so many circumstances," Dr. Magendie adds: "I speak of substances injected into the veins, the effects of which should be equally prompt and energetic. For example, I have several times introduced opium, in large quantities (ten grains), into the veins of rabid dogs, without producing the least narcotic influence; whilst a single grain of the aqueous extract passed into the veins of a dog in health, immediately produces sleep, which often continues for eight or ten hours. The same phenomena are observed in our own species. Mr. Dupuytren and myself have injected about eight grains of the watery extract of opium into the crural vein of a young man having hydrophobia, without any apparent result.* Rabid dogs have also supported the introduction of prussic acid in their veins, without experiencing an instant of relaxation in the progress of their disease.

"If substances as active as those above mentioned remain without effect, what hopes can reasonably be founded on vegetables which perhaps have no action on the animal economy, even in the state of health?"

These and other reflections led Dr. Magendie to attempt some new experiments, and to put in practice the following one. A large dog, of the mastiff kind, which had been decidedly mad at least two days, was muzzled and tied down on a table.

"I laid bare the jugular vein," says Dr. Magendie, "and commenced by taking blood from it to the extent of about a pound; after which I introduced, by means of a syringe, about sixty ounces of water, at the temperature of 40° centigrade, (105° Fahrenheit.) Towards the middle of this injection, the vessels were much distended; so, reckoning from this moment to the end of the injection, I suffered the blood to flow from the superior extremity of the vein, while I continued to introduce the water by the inferior. There escaped, in this manner, about ten or twelve ounces of blood mingled with water.

"The injection being concluded, I had the dog carried to his place, taking the greatest precautions; but these had become useless: the animal was calm; he laid down in a circle as it were to sleep, as soon as he was left to himself, which he had not done since the commencement of his disease. He no longer growled; his eye had become calm, and there was nothing menacing in his aspect: he only showed his teeth when attempts were made, by a long stick, to take away the straw on which he laid. I watched him for an hour; after which I went away, leaving one of my assistants to take charge of him.

"When I had terminated this experiment and had time to reflect respecting its consequences, some degree of regret accompanied the

* Dr. Breschet and myself inoculated a dog, under the skin of the forehead, with the saliva of this patient, and the animal became rabid at the end of about a month. Two dogs, who were bitten by this, also became mad after forty days. The latter bit several other dogs, but without any ill consequences.

pleasure I felt on seeing the symptoms of the disease so perfectly calmed: I feared that I had introduced too large a quantity of water into the veins. I knew that, if the injection had been carried too far, laceration of the pulmonary vessels would take place, and the animal would die from an infarction of the lungs, similar to that which so often causes the death of man. However, I was a little disposed to hope that the great size of the animal would permit his blood-vessels to contain, without bursting, so large a quantity of fluid. But it was not so: to my great regret, my assistant came, in the course of the day, to tell me that, in about five hours after the injection, the animal began to suffer great difficulty of respiration, which continued to increase for half an hour, when he died. To this time the dog had been very calm; he had not growled, and he had continued to lie as it were in a state of quiet sleep."

"The dog was dissected with the utmost care. No lesion of the brain, or of the spinal marrow, was found; the digestive organs were also in the healthy state, except that the salivary glands were red and appeared to be more voluminous than ordinary. The lungs were in a state of infarction; that is to say, their tissue was filled with aqueous blood: the bronchiæ and the trachea were filled with a brownish spume; their mucous membrane seemed to be inflamed."

---

Some curious and very interesting observations have recently been published by Dr. Kerner, of Wurtemberg, respecting the probable existence of a species of animal poison not hitherto known. Dr. Kerner says that the smoked sausages, which are so favourite a food with the inhabitants of Wurtemberg, often cause fatal poisoning. The effects of the poison occasionally manifest themselves every spring, in the month of April, in a more or less alarming manner. He states, that of 76 persons who became sick from having eaten those sausages, 37 died in a short time, and several others remained ill for years. The sausages made with liver appear to be the most dangerous. He is about to publish a tract on this subject. At present he remarks, that the poison which is generated in raw, minced, and seasoned flesh, smoked after having been stuffed in membranes, &c. is different from most others in not affecting the brain and the spinal marrow; whilst its influence is exerted on the lymphatic system, and sometimes the patient does not feel his heart beat for several months, although the pulsation of the arteries is unaltered.

Mr. Cadet, of Paris, says that he has been required by the police authorities, five or six times within the last fifteen years, to analyze meats bought at the sausage-shops at Paris. Those meats had proved poisonous, according to the statements of several physicians, and every means was taken by the legal authorities to discover the cause of these results. Mr. Cadet analysed all the meats; examined all the vessels in which they had been prepared; analysed the matters vomited, or found in the stomachs of the patients after death; without being able to find any trace of mineral poison by any of the tests in use. There was not an atom of copper, arsenic, or antimony; nor any evidence of malevolence or negligence. The shopkeepers were never brought into a court of justice; but similar accidents have recurred from time to time at Paris. The police officers have visited the pig-dealers, and have been satisfied that the pigs were fed with nothing but wholesome substances; the use of poison for rats, with which animals the places abounded, was interdicted, and every precaution taken that could be suggested. "What then," adds Mr. Cadet, "is this poison which is found in sausage-meats? Is it prussic acid? Is it a new matter? It is not the result of decomposition, or at least of putrefaction, because it exists in meats perfectly well preserved."

---

*To the Editor of the Medical and Physical Journal.*

MY DEAR SIR,

You are aware of the circumstances attending the transmission to Mr. Brande of the annexed Reply to an Article contained in his Journal for January last, purporting to be a review of my work on Prussic Acid. The result of my application to that gentleman for the insertion of this Reply in the next Number of that Journal, will be best shown by the following laconic correspondence.

*Saville-row, Wednesday evening, 7th February.*

*Dear Sir,*

*I send you a Reply to the Article contained in your last Number, purporting to be a review of my work on the Prussic Acid. Dr. Hutchinson, a particular friend of mine, has been kind enough to undertake to deliver it into your own hands, to prevent mistakes; and he is instructed to request you will have the goodness to name an hour in the evening of to-morrow, when he may call for your decision as to whether you will admit, or not, the said Reply for insertion in your next Number. In case of your declining to insert it (a circumstance which I am far from contemplating), I have requested my friend to bring back the MS. without any further comment.*

*I have the honour to be your humble servant,*

*A. B. Granville.*

*W. T. Brande, Esq. F.R.S.*

Two days afterwards, Mr. Brande returned me the manuscript of my Reply, with a short note, of which the following is the beginning:

*Thursday, (not delivered till Friday night.)*

*My dear Sir,*

*You must surely be quizzing me to suppose that I should insert the inclosed, &c.*

*I am always yours, faithfully,*

*W. T. Brande.*

*Dr. Granville, Saville-row.*

How far, by the refusal of an act of justice and impartiality demanded of him, and by the language in which that refusal is conveyed, Mr. Brande has or has not identified himself with my reviewer, and thereby rendered himself obnoxious to all and each of the charges I

have brought forward, and *proved*, against the latter,—I leave you to determine. It is enough for me to observe, that Mr. Brande's conduct as the Editor of the Quarterly Journal, in this affair, is to me a matter of great astonishment; and that, as a member of the Royal Institution, I shall take the earliest opportunity of protesting, either at a general meeting, or to the board of managers, against that Society lending its name to a Journal, in which an attack, involving matters of personal consideration, is admitted; and the reply, showing the injustice and unfairness of that attack, rejected.

Under these circumstances, you will perhaps allow me the opportunity of giving publicity to my Reply through the medium of your Journal; and I remain, my dear Sir, yours, &c.

A. B. GRANVILLE.

*Saville-row; Friday evening, 9th February.*

*A Reply to an Article inserted in the 20th Number of the Quarterly Journal of Science, edited at the Royal Institution; purporting to be a Review of Dr. Granville's Treatise on the internal Use of the Hydrocyanic Acid.*

In a Letter to W. T. Brande, Esq. F.R.S. the Editor.

DEAR SIR,

You will readily remember, that, when you informed me of having received a "severe" critique on my work on Prussic Acid for insertion in your Journal,—of which, however, you assured me that no use would be made; if I no longer entertained the opinion I expressed respecting the acid prepared at Apothecaries' Hall,—I instantly replied, that you were welcome to admit and insert the article in question, if you thought proper; for, as my opinion of the acid prepared at the Hall, at the specific period at which I was writing, had been formed upon such ocular and experimental demonstration as would have warranted still stronger expressions of disapprobation on my part, I could not tamely surrender my humble judgment to the terror of any review of my book, however severe. The only concession I claimed in return was, that any reply that I might think it necessary to write to the article you mentioned should be equally honoured with insertion in your Journal; and to the justice of this claim you readily acceded.*

In order to bring these circumstances more particularly to your memory, it will be well to mention that the conversation above referred to took place on the evening of the 14th of December, 1820, at Somerset House, a short time before the meeting of the Royal Society; and that it was again repeated by you, in a more cursory, yet impressive, manner, immediately after the Society had adjourned, as we were both in the act of leaving the meeting-room.

Had the review been 'severe,' yet just, correct, and candid, I should have preserved a becoming silence, and endeavoured to profit by the admonitions bestowed on me, however harshly; but, as it possesses none of the latter qualities, I can scarcely be expected to suffer it to pass unnoticed.

When I state that the review is the reverse of being just, correct, and candid, I am advancing what, I trust, I shall fully prove to yourself and the public; and this circumstance induces me to declare, *in limine*, that although, by designating that review as "severe," you implicitly acknowledged that you had read it, yet, from my acquaintance with your character and urbanity of manners, I am free to assume that you had not paid any very particular attention to the structure of that article, and the assertions it comprises; or it would never have appeared in your Journal, to which I am proud in having been one of the earliest, and certainly not the least zealous, of its contributors.

My observations, therefore, can in no way whatever apply to you as the editor of that Journal, except where it is expressly so stated; but are directed to the writer, of whom the letter O stands as the representative.

To render my reply as perspicuous and as concise as the nature of the subject will admit, I shall, whenever I have an opportunity, adopt the form of *positive answer* to the assertions of the reviewer rather than argumentative discussion, for which I have neither the necessary talent nor any very particular inclination. No answer or reply will be given to which I am not prepared to append a proof in support of its meaning; this being, in my opinion, the surest mode of conducting a defence.

The circumstances which induced the reviewer to notice my work are stated by him to be these:—

1. "The first part of it (the work) affects scientific arrangement; and the subject of which it treats *was first brought before the British public* in this Journal.

2. "We wish to point out an *error or two* into which the Doctor has fallen.

3. "And to advertise him of *two or three samples of bad taste* which have probably escaped his notice."

To which I then reply, *seriatim*,

REPLY 1.—The purely scientific part of the subject of Hydrocyanic Acid was *not* first brought before the British public in the Quarterly Journal of Science; neither was its application to the purposes of medicine first adverted to in that Journal.

*Proofs.*—A masterly account of the discoveries and investigations of Gay-Lussac on that subject was given in the Annals of Philosophy for December 1815, at which epoch the Quarterly Journal was *not* in existence! And a paper *specifically written for the British public*, respecting the use

* The reader will have seen, from Mr. Brande's reply to my letter given above, in what manner he has redeemed his implied pledge.

of Prussic Acid as a medicine, was inserted in the Medical Repository two years and a half before the subject was noticed in the Quarterly Journal.

*Corollary.*—O's first asseveration, therefore, is 'incorrect.'

REPLY II.—Of the one or two errors into which I am said to have fallen by the reviewer, *one only* is mentioned, after all, by him in the course of his critique, at page 402, respecting the specific gravity of prussic acid; and that is *not* an error of the author, but a typographical fault.

*Proof.*—See the *errata corrige* prefixed to my work, in which that fault is actually rectified.

*Corollary.*—O's first accusation against me, therefore, is 'unjust.'

REPLY III.—Of the two or three samples of bad taste of which O was anxious to 'advertise me,' one only is brought forward by him in his review; and that one sample of bad taste is only made to appear as such by artfully coupling together two short garbled quotations from my book.

*Proof.*—The anecdote related in my book, from which the quotations of the reviewer are taken, relates to a matter of fact, which he has not dared to repeat; or what he has called 'bad taste' would have appeared to be 'plain truth.'

*Corollary.*—O's conduct, therefore, is 'wanting in candour.'

After sketching a short and rapid account of the history of prussic acid, taken in some instances *verbatim*, though without acknowledging it, from my work, the reviewer proceeds to assert (page 401) that—

1. I have adverted to the history of that substance *superficially*.

2. That I have given the different processes for preparing the acid *without sufficient remarks upon their principles*.

3. That I have passed judgment upon the merits of those processes, *not always tempered with mercy*.

To which assertions I beg to apply the following answers:

ANSWER I.—The chemical history of Prussic Acid is *not* adverted to superficially in my treatise. It is more fully given than in many recent works on chemistry. It is a hundred times more extended than that substituted by the reviewer for the edification of his readers.

*Proofs.*—My chemical history of Prussic Acid begins from the discovery of Prussian blue, and terminates with the latest researches respecting the component parts and real nature of the acid itself, by Gay Lussac, whose notions and atomic theories are fully given; while the intermediate epochs of this interesting history are duly noticed; the labours of several eminent chemists, particularly those of Morveau, Scheele, Berthollet, &c. as well as those of M. Porrett in this country, are mentioned; and continued reference made throughout the section, as well as at the conclusion of it, to various works in which the subject has been treated in the most satisfactory manner. The historical account above alluded to occupies *twenty-four* printed pages of my work; while not one-third of that space, in the most approved modern works on chemistry, has been dedicated to that subject, excepting in the laborious and classical System of Chemistry by Dr. Thomson. The chemical history of the same substance substituted by the reviewer himself, occupies just *twenty-nine* lines.

*Corollary.*—O's assertion, therefore, respecting my *superficiality* is incorrect.

ANSWER II.—It is *not* true that I have given the different processes for preparing the acid 'without any sufficient remarks upon their principles.'

*Proofs.*—My description of Scheele's process, written with what perspicuity I could master, is followed up by a complete *rationale* of the various steps of that process, which had been ambiguously interpreted by others. Of Vauquelin's process I observed, that its simplicity would render any remark of mine upon it an act of supererogation; and on the third, or Magendie's process, consisting in the mere dilution of the concentrated acid with water, I made enough remarks to attract the notice of the reviewer, who has grounded upon them a whole and long paragraph concerning their pretended incorrectness!

*Corollary.*—O's assertion, therefore, is again in this instance 'unjust.'

ANSWER III.—The judgment I passed on the different processes, is so far from being 'not tempered with mercy,' that in two cases it is given in a strain of eulogium; and in the third no judgment is given at all!

*Proofs.*—Speaking of that of Scheele, I state, 'By this method the acid is obtained at an uniform degree of concentration;' and again, 'this acid is perfectly good for the purpose of practice;' while, on the subject of that of Vauquelin, I affirm 'that the acid prepared according to his method is of a proper strength for medicinal purposes.'

*Corollary.*—O's assertion, therefore, in this case, is again wanting in candour.

And here I cannot help observing to you, my dear Sir, that this advocate for mercy so far forgets his own precepts, that, throughout the review of my work, sentiments are uttered, and expressions used, that are very distantly allied, indeed, to that heaven-born virtue. As a proof of this assertion, I need only mention that the very processes of two such eminent chemists as Scheele and Vauquelin, which he affects to defend from my unmerciful judgment, are by him dismissed in the most peremptory language of condemnation; the one as furnishing an acid of 'variable composition;' the other for being 'extremely objectionable.'

Speaking of the very curious but difficult branch of chemical inquiry respecting the formation of prussic acid by the combination of animal matter, contained in the third section of my book, the reviewer says that 'I should either not have meddled with it, or given a clear epitome of what is known upon the subject.' In answer to which I have to observe—

1st. That I have *not* meddled with the subject, which is still involved in absolute obscurity, any further than by repeating an uncontroverted fact, which, instead of being quoted in a garbled manner, should have been fairly transcribed by the reviewer; and, 2dly. That the whole of what is known on the subject of the formation of prussic acid by the combination of animal matter, *is* detailed in the said section, although that *all* be but little, and betray 'a poverty in the land,' as the reviewer poetically expresses it. And I challenge him to point out any book on chemistry in which more, or even as much, is to be found on that subject, that is not conjectural.

*Proofs.*—The very paragraph with which the section in question of my work begins, and which the reviewer imperfectly quotes as unintelligible, is given in the identical language of Berthollet, from whose *Essai de Statique Chimique* it was collected. Neither Thomson, nor Murray, nor Henry, nor Klaproth, nor Orfila, nor Lagrange; no, nor even yourself, in your manual; have alluded to the inquiry in question in the slightest degree: so that the 'epitome of what is known' may be well comprehended in the two pages that I have dedicated to that subject.

*Corollary.*—The reviewer, therefore, is on the present occasion, as on all the preceding ones, wanting in justice as well as candour.

But I have done more. I have given an extract from my notes taken while attending a course of lectures on animal chemistry delivered by Vauquelin, in which an endeavour is made to place the above subject in a somewhat clearer light. Yet what is the conduct of O on this occasion? With the same *nonchalance* with which he had just before talked of the *unintelligibility* of two paragraphs borrowed from Berthollet and Thenard, he assures his readers that the extract from Vauquelin's lectures is not a tittle more intelligible; and dismisses it without any quotation in support of his affirmation. Now, as I mean, throughout this letter, to substantiate by *proofs* what I advance on the score of O's candour and justice; and as I hesitate not to assert that the charge of unintelligibility against Vauquelin, is as gross a defection from truth as that which characterizes the charge brought against his process for preparing the hydrocyanic acid, I shall beg leave to quote once more the passage in question, in order that the reader may judge whether or not it be *unintelligible*. 'When animal substances are exposed to heat with a mixture of alkalies, hydrogene, carburetted, and carbonic gas, are obtained, besides a residuum, which, if washed in water, will be found to contain prussic acid. The alkali therefore seems necessary to form the prussic acid, by attracting together the principles of which it is constituted,'* If this be unintelligible, then plain language is not capable of expressing common ideas; but if, on the contrary, the passage be found perfectly comprehensible, and such, indeed, as will be met in substance in the works of most men of eminence who have written on the same subject, then the conclusion regarding the reviewer's candour is unavoidable.

The reviewer's charge (at page 406) of my having unnecessarily separated the account of the physical properties of the prussic acid from its chemical history and preparation, is of too trivial a nature, and absurd, to need refutation. Every book on chemistry is full of examples of such practice; and Gay Lussac himself has followed no other method in his admirable essay on Hydrocyanic Acid.

In the succeeding paragraph of his critique, relating to the physiological experiments made with the *pure* hydrocyanic acid by several authors and myself, the reviewer remarks, that, 'as Mr. Brodie's investigations *upon this subject* are the most satisfactory that have hitherto been made, and as they are not even alluded to by me, he shall decline troubling his readers with those I have detailed.' To which this is my reply:

1. Mr. Brodie *never* made any experiment with the pure hydrocyanic acid.

2. Previous and subsequently to Mr. Brodie's investigation respecting the action of various poisons on animals, Coullon, Emmert, Magendie, and others, had and have instituted experiments with the pure hydrocyanic acid, or with substances containing it, not only upon animals, but upon the human system; which, in a work of practical utility, and not simply of philosophical speculation, could not but be preferred to every other experiment.

Either, therefore, O knew all this; and, in such a case, where is candour and truth in concealing it?—or he knew it not; and in that case it was his duty, ere he undertook to criticise the book, to have made himself master of its subject.

The eighth section of the work relates to the means of detecting prussic acid, and preventing its poisonous effects: 'in neither of which,' says the candid and just reviewer, 'do we remark any thing either very new or very important;' but respecting which I must beg leave to ask him two questions.

1. Is it not *very important* to determine the symptoms of poisoning by this acid, and to ascertain the best means for counteracting its deleterious effects? These objects have been accomplished, as far as they could be, in the said eighth section.

2. Is it not *very important* to be acquainted with the means of detecting the presence of prussic acid, particularly in cases of death from that substance? And have these means, or the mode of conducting the investigation, been pointed out to the public before the appearance of my work, by any chemist, English or foreign? Is not that new, which is not to be found elsewhere? Is any thing of the kind contained in the works of Fourcroy, Chaptal, Thenard, Thomson, Murray, Orfila, Henry, Children, or even in your own Manual of Chemistry? No.

Then what becomes of the justice, correctness, candour, and, I may now add, the knowledge (in these matters) of the reviewer? Perhaps some evidence of all these qualities is to be found in the remaining part of the Review, relating to the most important as well as to the largest portion of my work, which is dismissed in ten lines and a half!

I am now arrived at that part of my reply upon which I enter with feelings of great reluctance; because it alike involves charges of a heavy nature against the reviewer, and obliges me, from a sense of what is due to truth, publicly to deny the correctness of opinions said to be your own.

My charges against the reviewer, are, 1. Misrepresentation, or concealment of facts. 2. Ignorance of the subject on which he has undertaken to pronounce. 3. Unworthy insinuations against the author whose book he reviews. Each and all of which charges, in pursuance of the plan I have followed throughout this Reply, I shall proceed to substantiate by positive proofs; leaving, however, to the public, the task of drawing, in this instance, the corollaries that must necessarily follow.

The first charge, or that of misrepresentation or concealment of facts, is supported by the following evidence:—1. The reviewer tells his readers that the formula of Dr. Magendie for diluting Gay Lussac's acid is not given in my book, (p. 402;) whereas the *fact* is, that, at p. 20 of my Treatise, I *have inserted* that formula thus: 'Dr. Magendie dilutes the concentrated acid of Guy Lussac with six times its volume, or eight times and a half its weight of distilled water.'——2. The reviewer, in the same paragraph, informs his readers that the number 9·20583 is quoted by me as the 'medium density' of Magendie's diluted acid: whereas the truth is, that I distinctly used the word *weight*, meaning the absolute weight, and not the *medium density* of a mixture of 8·5 of water and 0·70583 of concentrated acid.——3. The reviewer says, I have 'fallen into some sad errors respecting the specific gravity of the pure acid;' and merrily and triumphantly quotes a typographical fault, by which I am made absurdly to state the specific gravity of the acid to be 70·583; whereas in the *errata*, which every candid reviewer would have turned to on seeing such an absurd mistake, the printer's error is actually rectified, and the specific gravity correctly given thus—·70583.——4. The reviewer states, 'that the Doctor insinuates, though he must know better, that the acid sold at Apothecaries' Hall is *always* turbid, yellowish, and *impure*.' This is *not* true. The Doctor never expressed such an insinuation; nor did he use the two words, 'always' and 'impure,' which the reviewer, with utter disregard to propriety, has attributed to him, and has even marked in italics.

* It is not a little curious that one of the most important works on Chemical Science written in the English language, should contain a passage nearly similar in import to the above, on the subject of Prussic Acid. Having described the process of heating blood and alkalies to procure the acid, the author proceeds to give the following explanation of that process:—'This process consists essentially of two operations; one, the impregnation of the alkali with that peculiar principle contained in the blood, which gives the power of striking a blue colour with iron, and is called the prussic acid,' &c.—*Vide Aikin's Dictionary of Chemistry, art.* Prussian Blue.

The following is the only passage in which I passed any degree of condemnation on the acid prepared at Apothecaries' Hall: ' I know, besides, that the acid thus prepared is of a turbid yellowish colour, instead of being colourless and transparent, and that it deposits a considerable sediment; both which circumstances *seem* greatly to militate against its *purity*.'

The second charge against the reviewer, or that of ignorance of the subject on which he has undertaken to pronounce, is thus substantiated. 1. The reviewer states, at p. 400, that he is 'not quite clear' whether Gay Lussac gave the name of *cyanogene* to the base of hydrocyanic acid, ' because it burns with a bluish-purple flame, or because it is essential to the production of Prussian blue;' whereas Gay Lussac is positive as to the latter reason; and every chemist acquainted with the subject knows it to be so.——2. The reviewer asserts, that the acid obtained by Scheele's method ' is of variable composition, because Prussian blue is not always of equable purity;' but this is not true in practice. Mr. Garden has prepared prussic acid according to this method for the last three years and a half, with an invariable precision of result and equality of strength, which, to judge of its constant specific gravity, is stronger than that prepared at Apothecaries' Hall.——3. The reviewer has acknowledged his inability of obtaining *pure* prussic acid by Vauquelin's method, which he asserts to have tried frequently. But the want of success in his case must be ascribed to his ignorance in chemical manipulations, *as you well know;* for you *have seen* pure prussic acid prepared according to that method.——4. The reviewer says he cannot understand the following passage of my work, containing the well-known hydrodynamic axiom, that ' the weight of fluids is equal to their volumes multiplied by their densities.' I can only observe in reply, that, if he is a stranger to mathematical language, he has only to refer to Dr. Young's Lectures on Natural Philosophy, or Biot's, or Brisson's, or any other author's, work on the same subject; in all of which that very axiom is mentioned and explained. In the instance above alluded to, the passage was translated *verbatim* from a note in Dr. Magendie's pamphlet on the subject of which I was commenting.——5. The reviewer, making himself quite merry, and rioting in the pleasure of the discovery of ' the Doctor's sad errors,' asserts that the specific gravity of a mixture of six volumes of water at 1, and one volume of acid at 0·70583, such as Magendie employs, *must be* 0 9900; whereas any person acquainted with the common rules of alligation, would know that $\frac{6\times1+0\cdot7853}{7}=0\cdot9579$. To which I add, that such is nearly the actual specific gravity of the mixture in question, ascertained by repeated experiments; the ' increase of density resulting from the mixture of the pure acid with water' not being so ' great' as the reviewer boldly asserts.

The third charge against O, namely that of throwing out unworthy and unwarrantable insinuations against me, will be best substantiated by a quotation of his own expressions. ' We should have conceived it more decorous on the part of Dr. Granville, finding the above preparation objectionable, as he has asserted it to be, to have stated the objections to the Apothecaries' Company, *instead of publishing their process with a view to depreciate it, and to employ it as a vehicle of a puff-oblique in favour of the doctor's chemist, Mr. Garden*." I despise too much the individual, who, without the slightest degree of evidence in support of them, can assume and publish two such inferences, and direct them against the moral rectitude of an author—to be disposed to take any other notice of the above disgraceful insinuations against my character, than to express my utter astonishment, that you should have suffered it to appear in your Journal! Did you know of any thing in my conduct during the six or eight years of our acquaintance; or any passage in my work which could have led you to admit, for one instant, the propriety of the aspersions of my reviewer? The only passage that relates to Mr. Garden, the chemist, and which is contained in the same paragraph of my work, which seems to have given such mortal offence to O, is this " I have not had an opportunity of trying this acid (the apothecaries'), as I am satisfied with that which Mr. Garden prepares for my patients." Is *truth*, then, synonimous with *puff-oblique* in the moral lexicon of my reviewer?—As to its being or not " *more decorous*" for me to have stated the objections I entertained against their acid, to the Apothecaries' Company themselves, instead of publishing those objections; I have yet to learn that that worshipful body have any claim to the services of any physician. Let them look to their own business, and see that their own officers do their duty. O gives a sort of manifesto in his review, from the Apothecaries' Company, in which they declare, that they "*have no secrets;*" if so, why should he feel sore at my having published their process of preparing the hydrocyanic acid? and how, I would furthermore ask him, can the publication of such a process, " *depreciate it*," as he rather aukwardly states, if the process be inherently perfect?

A few more words on the subject of certain opinions ascribed by the reviewer to yourself; and on the defence set up by that gentleman in favour of the apothecaries' acid of 1819 and 1820, and I conclude.

You are said in the Review, to have reported to the Laboratory Committee of the Apothecaries' Society that, ' having tried the methods of Scheele and Vauquelin you found them uncertain in their products, and more especially in the latter case, the specific gravity of Vauquelin's acid *always* exceeding that of distilled water.' In opposition to which opinions, I assert, 1. That Scheele's method, with common precaution, yields, by far, the most equable and the purest acid for medicinal purposes, of a specific gravity inferior to that quoted by you as the density of the acid prepared according to your own formula, and which is stated to be 0·995, although I have found it to be as high as 0·998 in two specimens procured at the Hall a few weeks back—whereas, 0.993 is the invariable density of Scheel's acid at an uniform temperature. 2. That by Vauquelin's method, prussic acid, possessing all the requisite physical and medicinal properties, colourless, transparent, and of the specific gravity of 0 998, and, consequently, *under the standard density* of distilled water, can be procured—is annually procured to a large amount by the French chemists, who sell no other—and has been procured by me at two different times, since your assertion to the contrary has been published by the reviewer. You have not, I suppose, forgot, that on the evening of the 18th ultimo, I shewed you, at the table of the Royal Society, a specimen of the acid so prepared by me, as well as another prepared, according to Scheele's process, by Mr. Garden, which you admitted to be ' as good specimens as could be desired, and the purity of which, to judge of their specific gravity marked on the phials containing them, seemed to be quite unobjectionable.' It was on that same occasion, that I remarked to you, that the accuracy of Vauquelin was too well established, to allow us for a moment to suppose, that he would have recommended a process which, according to your expressed opinion, ' is uncertain in its products,' and the specific gravity of which ' is *always* greater than that of distilled water.' To the justice of this remark you then assented. The specimens alluded to were shewn that same evening, and in the same place, to Dr. Holland and Messrs. Phillips and Faraday, to whom, as well as to you, I exhibited two other specimens of the acid, procured at Apothecaries' Hall in 1819 and 1820. One of these, presented a fluid of a dark-brown colour—turbid when shaken—but transparent when suffered to rest so as to give time to a copious blackish sediment to subside: the other offered a fluid of a muddy coloured appearance, though by no means so striking as in the former case. From these two specimens, obtained at two different periods from the Hall, I then declared to you, and to the other gentlemen above mentioned, what I again repeat on the present occasion, that the description of the Apothecaries' acid, contained in the second edition of my work, was taken; and I may, I think, call upon you to say, whether under such circumstances, I should not have been justified in passing a *severer* judgment upon the merits of that acid, than that which my reviewer has qualified by the expression, 'not tempered with mercy ;' and to which I omitted to advert in another part of this reply, because I considered the present as a fitter opportunity for so doing.

The fact is, that both you and the reviewer—the one verbally, in conversation with me—the other in writing, at page 404 of his Review, have admitted the justice of my remark as to the appearances of the acid prepared at Apothecaries' Hall; and it remains only for me to say, in conclusion, that if my reviewer can assert as he has done at page 404, that 'the occasional yellowness and turbid appearance of that acid is *rather an indication of its purity than otherwise*,' his logic will certainly fail to convince his readers, as it failed to convince myself. If, however, by the admission that the acid is occasionally yellow and turbid, and presents moreover, a sediment, its decomposition is also admitted (which the reviewer has virtually done); then a preparation liable to such fatal objections, ought not to have been defended as a 'very uniform and very pure product;' nor sold as *pure prussic acid* by the Apothecaries' Company, to supply 'the occasional demand' for that article, as they have unquestionably done in the case of the two specimens in my possession, which were nearly as objectionable in their appearance when first procured, as they are now, when 'age' and 'purity,' says the reviewer, are to be considered as the two causes of those objectionable appearances. For the information and caution of every medical practitioner, I publicly exhibited, last night, at a meeting of the Medico-Chirurgical Society, the two latter specimens, as well as those alluded to in the course of this letter; which have been prepared agreeably to Scheele's and Vauquelin's methods—and will shew them to any other member of the profession, who may be desirous of inspecting them.*

As to the tone and style, generally, in which O's review is written, I can only assent to the judgment passed upon them, in a scientific circle, by one of the first philosophers in the country;† to whose opinion both you and I must readily bow—which judgment, given in the presence of two or three other gentlemen, but addressed to me in particular, went to condemn both as equally derogatory from the dignity of a reviewer, and injurious to the journal in which such a Review has been admitted.

I have the honour to be your humble servant,

A. B. GRANVILLE.

*Saville-row, Wednesday, 7th Feb.* 1821.

---

* The relative value of the acid prepared by Mr. Garden, and at the Hall, agreeably to Mr. Brande's formula, will be further illustrated by the following facts. A pupil of St. Bartholomew's Hospital assured me, the other day, that the Apothecaries' acid had been administered to patients in that hospital in doses of twenty-four drops at a time, without the slightest obvious effect. Mr. Travers mentioned, at the Medico-Chemical Society, a few nights since, that a servant of St. Thomas's Hospital having inadvertently swallowed a mixture containing from ten to twenty drops of acid, which he mistook for an aperient draught, fell down on the floor, as if shot by a cannon-ball, and continued it for several days. The acid used in this hospital is prepared at Mr. Garden's.

† This last paragraph has undergone a trifling verbal alteration, since the manuscript was submitted to Mr. Brande, and rejected—in consequence of that gentleman having erroneously interpreted its original meaning, and with a view to avoid any such misinterpretation.

---

### MONTHLY CATALOGUE OF MEDICAL BOOKS.

### METEOROLOGICAL JOURNAL.

By Messrs. William Harris and Co. 50, Holborn, London.

*From January 20 to February 19, inclusive.*

| Day of Month. | Moon. | Rain gauge | THERM. | | | BAROM. | | DeLuc's Hygro. | | WIND. | | ATMOSPHERIC VARIATION. | | |
|---|---|---|---|---|---|---|---|---|---|---|---|---|---|---|
| Jan. | | | | | | | | | | | | | | |
| 20 | | | 48 | 50 | 40 | 30·35 | 30·53 | 62 | 62 | WSW | W | Fine | Cloud. | |
| 21 | | | 38 | 42 | 39 | 30·63 | 29·65 | 60 | 60 | W | SW | Fog | Fine | Cloud. |
| 22 | | | 40 | 42 | 38 | 30·62 | 30·62 | 60 | 60 | W | WNW | Cloudy | | |
| 23 | | | 40 | 41 | 38 | 30·72 | 30·73 | 60 | 60 | NE | NE | Cloudy | Fine | Cloud. |
| 24 | | | 39 | 41 | 33 | 30·67 | 30·61 | 59 | 61 | NE | N | Fog | Cloud. | Fine |
| 25 | | | 38 | 42 | 38 | 30·59 | 30·57 | 62 | 63 | NNE | SE | Fog | Cloud. | |
| 26 | ☽ | | 39 | 41 | 38 | 30·57 | 30·50 | 62 | 60 | NE | ENE | Cloudy | | |
| 27 | | ·03 | 38 | 40 | 36 | 30·40 | 30·32 | 60 | 63 | ESE | SE | Fog | Cloud. | Rain |
| 28 | | ·05 | 37 | 39 | 33 | 30·25 | 30·27 | 61 | 60 | S | SSW | Rain | Cloud. | Fine |
| 29 | | | 36 | 38 | 38 | 30·21 | 30·25 | 59 | 62 | SSE | SSW | Fine | Fine | Cloud. |
| 30 | | | 45 | 48 | 42 | 30·30 | 30·30 | 63 | 65 | SSW | SW | Cloudy | Fine | |
| 31 | | | 46 | 49 | 45 | 30·37 | 30·40 | 64 | 64 | SW | WSW | Fine | | |
| Feb. | | | | | | | | | | | | | | |
| 1 | | | 49 | 50 | 45 | 30·39 | 30·25 | 63 | 63 | SW | W | Cloudy | Fine | |
| 2 | ● | | 47 | 50 | 37 | 30·20 | 30·27 | 63 | 60 | WSW | SW | Cloudy | Sho'ry | Fine |
| 3 | | | 41 | 45 | 35 | 30·32 | 30·17 | 58 | 58 | WSW | WSW | Fine | | |
| 4 | | | 38 | 42 | 32 | 30·21 | 30·33 | 57 | 58 | WNW | NNE | Cloudy | | Fine |
| 5 | | | 33 | 38 | 32 | 30·61 | 30·76 | 57 | 55 | N | SW | Fine | | |
| 6 | | | 35 | 41 | 32 | 30·76 | 30·71 | 54 | 53 | SW | SW | Cloudy | Fine | |
| 7 | | | 35 | 43 | 34 | 30·70 | 30·68 | 54 | 54 | SW | SW | Fine | | |
| 8 | | | 38 | 45 | 33 | 30·58 | 30·42 | 56 | 55 | SSW | SE | Fine | | |
| 9 | ☾ | | 34 | 42 | 36 | 30·22 | 30·10 | 54 | 57 | SE | NE | Fog | Fine | |
| 10 | | | 40 | 45 | 35 | 30·25 | 30·46 | 60 | 60 | NE | ESE | Cloudy | Fine | |
| 11 | | | 37 | 42 | 34 | 30·34 | 30·25 | 57 | 57 | E | E | Cloudy | | |
| 12 | | | 34 | 38 | 34 | 30·24 | 30·31 | 58 | 58 | NE | NE | Cloudy | Fine | Cloud. |
| 13 | | | 35 | 38 | 34 | 30·31 | 30·25 | 57 | 57 | NE | E | Cloudy | Fine | |
| 14 | | | 35 | 38 | 34 | 30·23 | 30·31 | 57 | 57 | NE | WSW | Fog | | Cloud. |
| 15 | | | 34 | 38 | 31 | 30·36 | 30·50 | 57 | 57 | WNW | E | Fog | Cloud. | |
| 16 | | | 33 | 36 | 31 | 30.51 | 30·42 | 56 | 57 | E | SSE | Cloudy | | |
| 17 | ○ | | 33 | 36 | 30 | 30·37 | 30·24 | 58 | 59 | NE | NNE | Fog | Cloud. | |
| 18 | | | 34 | 37 | 30 | 30·18 | 30·26 | 58 | 58 | NW | W | Fog | Cloud. | |
| 19 | | | 32 | 35 | 31 | 30·31 | 30·30 | 57 | 57 | NW | WNW | Fine | | |

The quantity of rain fallen in the month of January, is 2 inches and 9-100ths.

